# Poor self-reported adherence to COVID-19-related quarantine/isolation requests, Norway, April to July 2020

**DOI:** 10.2807/1560-7917.ES.2020.25.37.2001607

**Published:** 2020-09-17

**Authors:** Anneke Steens, Birgitte Freiesleben de Blasio, Lamprini Veneti, Amy Gimma, W John Edmunds, Kevin Van Zandvoort, Christopher I Jarvis, Frode Forland, Bjarne Robberstad

**Affiliations:** 1 Division of Infection Control and Environmental Health, Norwegian Institute of Public Health, Oslo, Norway; 2 Oslo Center for Biostatistics and Epidemiology, Department of Biostatistics, Institute of Basic Medical Sciences, University of Oslo, Oslo, Norway; 3 Centre for Mathematical Modelling of Infectious Diseases, Department of Infectious Disease Epidemiology, London School of Hygiene & Tropical Medicine, London, United Kingdom; 4 Department of Global Public Health and Primary Care, University of Bergen, Bergen, Norway; 5 Division for Health Services, Norwegian Institute of Public Health, Oslo, Norway

**Keywords:** COVID-19, quarantine, isolation, adherence, control measures, SARS-CoV-2

## Abstract

To limit SARS-CoV-2 spread, quarantine and isolation are obligatory in several situations in Norway. We found low self-reported adherence to requested measures among 1,704 individuals (42%; 95% confidence interval: 37–48). Adherence was lower in May–June–July (33–38%) compared with April (66%), and higher among those experiencing COVID-19-compatible symptoms (71%) compared with those without (28%). These findings suggest that consideration is required of strategies to improve people’s adherence to quarantine and isolation.

Quarantine of contacts and isolation of ill people are important control measures to limit the spread of severe acute respiratory syndrome coronavirus 2 (SARS-CoV-2) and thereby prevent coronavirus disease (COVID-19) [1]. Knowledge on adherence to these control measures is important to plan necessary public health interventions.

## Determining self-reported adherence to quarantine and isolation

We determined self-reported adherence to quarantine and isolation in a prospective cohort study among Norwegian adults 18 years of age and older. The study population included a population-representative random sample concerning age, sex and location recruited from a standing Internet panel [2]. Individuals who subscribe to the Internet panel are recruited through a variety of sources, including social networks, email lists, banner ads, specialised websites, co-registration and search engine marketing. Provision of moderate financial-like incentives for completing surveys contribute to targeting hard-to-recruit populations. Participants sampled from the panel to participate in our study were repeatedly asked to answer an online questionnaire in 3- to 6-week intervals.

**Figure 1:**
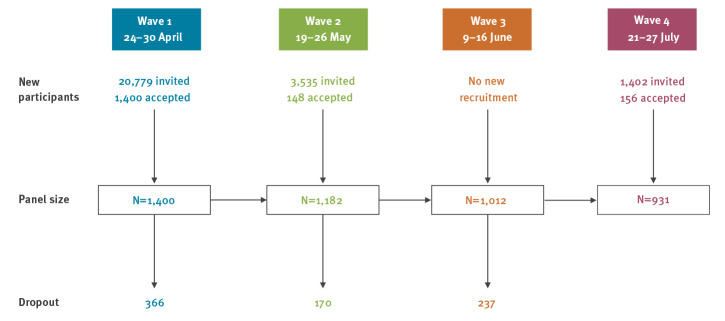
Flowchart of recruitment and dropout of COVID-19 quarantine/isolation study, Norway, April–July, 2020

We report here early results from the first four waves of data sampling covering the months April to July 2020; 1,400 participants were initially recruited (response rate: 7%). Because of dropout (response rate for subsequent waves: 74–86%), additional panel participants were recruited in successive waves (Figure 1).

Panel members were asked, among others, questions about demographics, symptoms and adherence to control measures. We evaluated adherence by jointly considering whether participants, during the previous 7 days (i) had been asked to quarantine or isolate themselves, and (ii) had been in quarantine or isolation for at least 1 day. In the questionnaire, quarantine and isolation were defined in the same way as in the official information given by the Norwegian health authorities [3]. Isolation is required in Norway only for those with confirmed or probable COVID-19. However, several participants reported having received a request to self-isolate or having isolated themselves while not having been tested (11%; n = 194 / 1,704). We are, therefore, uncertain whether the definitions of quarantine and isolation were understood correctly by all and pooled these variables into one variable ‘quarantine/isolation’ in the analysis.

Among those reporting quarantine/isolation request(s), we defined a person adherent when he/she had been in quarantine/isolation at least 1 day or non-adherent when not. People who reported requests in multiple waves can have both an adherent and a non-adherent status as we pooled the different waves within participants. For the analysis over time, participants could only have one status per wave. Because of the different frequency of participation and because not all participants reported having received quarantine/isolation requests each wave, the time trend has not been tested for statistical significance as these reasons for ‘missing data’ would severely decrease the power and would likely introduce bias.

Using the survey design in Stata 16.0, we weighted the analysis by age and sex to obtain results representing the Norwegian population; all presented percentages are weighted and provided with 95% confidence intervals (CI), unless mentioned otherwise. We used age/sex at time of inclusion to define weights for the overall results and wave-specific age/sex-weights for the analysis over time.

## Description of the participants

Overall, 1,704 individuals participated in at least one wave (Table 1). Participants were aged between 18 and 89 years (median 50 years), and 51% were male. The age groups 18–29 years and 50–69 years were slightly under- and overrepresented in our sample, respectively, compared with the Norwegian population.

**Table 1:** Description of the participants in COVID-19 quarantine/isolation study, at time of inclusion, Norway, 2020 (n=1,704)

Variable	Number	Percentage in this panel (unweighted)	Percentage in the Norwegian population [11]
**Frequency of participation**			
1 wave	453	27	NA
2 waves	296	17	NA
3 waves	340	20	NA
4 waves	615	36	NA
**Age group in years**			
18–29	243	14	20
30–49	577	34	34
50–69	650	38	30
70–89	234	14	16
**Sex^a^**			
Male	876	51	50
Female	827	49	50
**Region**			
Living in Oslo (where the epidemic hit hardest)	312	18	13
Living outside Oslo	1,392	82	87
**Risk group**^b^			
Self-reported medical risk group or pregnant	714	45	29 [12]
No medical risk group	876	55	71

NA: not applicable.

^a^ Total n=1,703; one answer in category ‘not applicable/other’ excluded from denominator.

^b^ Defined by the question ‘Are you in a high-risk group under which the annual influenza vaccine would usually be offered?’ This includes age ≥ 65 years [11,12]. Total n=1,590; 114 participants answered ‘unknown’.

## COVID-19-related symptoms and testing

In total, 40% (95% CI: 38–43, n = 671) of the participants reported at least once symptoms compatible with COVID-19 within the last 7 days of reporting (symptoms see footnote ^a^ Table 2). Nine percent (95% CI: 7–10; n = 142) reported testing at least once for SARS-CoV-2. This percentage was similar to the Norwegian population where 8.5% were tested by end-July [4]. Of tested study participants, 13% (95% CI 8–21; n=15, 4 results were pending) were SARS-CoV-2 positive.

**Table 2:** Quarantine/isolation request and adherence, overall and for those who reported to have had COVID-19 compatible symptoms, COVID-19 quarantine/isolation study, Norway, April–July, 2020

Variable	Reported symptoms^a^	Number ‘Yes’ / number with useful information^b^	Percentage ‘Yes’
**By events, independent of participation in several waves**^c^			**Unweighted (in sample)^d^**
Received a quarantine/isolation request	Overall	574 / 4,407	13
	Symptoms	174 / 1,092	16
	No symptoms	393 / 3,286	12
Quarantine/isolation events	Overall	417 / 4,467	9
	While reporting a request	204 / 561	36
	Symptoms	198 / 1,102	18
	No symptoms	217 / 3,333	7
Adherence events^e^	Overall	204 / 561	36
	Symptoms	116 / 170	68
	No symptoms	87 / 385	23
Non-adherence events	Overall	357 / 561	64
	Symptoms	54 / 170	32
	No symptoms	298 / 385	77
**By participant – pooled answers over all available waves**			**Weighted (extrapolation to the population) (95%CI)^d^**
Received a quarantine/isolation request	Overall	402 / 1,642	25 (23–27)
	Symptoms ^f^	125 / 648	21 (17–24)
	No symptoms ^f^	297 / 1,390	22 (19–24)
In quarantine/isolation at least 1 day in at least 1 wave	Overall	298 / 1,673	19 (17–21)
	Symptoms ^f^	148 / 657	25 (21–28)
	No symptoms ^f^	170 / 1,417	13 (11-15)
Adherence^e^ to quarantine/isolation in at least 1 wave	Overall	154 / 393	42 (37–48)
	Symptoms ^f^	85 / 124	71 (63–79)
	No symptoms ^f^	75 / 290	28 (23–34)
No-adherence to quarantine/isolation in at least 1 wave	Overall	270 / 399	65 (60–70)

CI: confidence interval.

^a^ Symptoms compatible with COVID-19 were: fever or high temperature, a cough that has lasted for at least several hours, shortness of breath, aches and pains (e.g. in back, neck, shoulders or joints), blocked nose, sore throat and feeling unusually tired.

^b^ Useful information means here answering yes or no. Those with missing information, those reporting ‘I prefer not to answer’ and those reporting ‘I do not know’ are excluded. See Supplementary table for all numbers.

^c^ Dependency of the data ignored in analysis.

^d^ Those who answered ‘Do not know/prefer not to answer’ were excluded from the analysis. Additionally, those reporting ‘No quarantine/isolation requests’ were also excluded from the adherence analysis.

^e^ Adherence was defined as ‘Received a quarantine/isolation request’ and reported as the percentage of ‘Was in quarantine at least 1 day’ during the last 7 days at the time of answering the questions.

^f^ The sum of those with symptoms and those without symptoms is more than the overall numbers. This is because a person can have different statuses in different waves.

## Self-reported quarantine/isolation requests and adherence

The 1,704 study participants filled out 4,525 questionnaires; complete information on quarantine/isolation requests and behaviour was available for 97% (4,385/4,525). Respondents reported 574 quarantine/isolation requests and 417 quarantine/isolation events (Table 2).

About 25% (95% CI: 23–27) of individual participants received at least one request to quarantine/self-isolate in the 7 days before responding (Table 2). Of these, 42% (95% CI: 37–48) reported to have at least once adhered to this request and 65% (95% CI: 60–70) reported not to have adhered to the request at least once. Adherence was substantially higher among those with COVID-19 compatible symptoms (71%; 95% CI: 63–79) compared with those without symptoms (28%; 95% CI: 23–34). It was also higher among those aged 18–29 years (72%; 95% CI: 58–83; n = 42) compared with those aged 30–49 (48%; 95% CI: 38–59; n = 43), 50–69 (24%; 95% CI: 18–32; n = 37) or 70–89 years (36%; 95% CI: 26–47; n = 32).

## Declining adherence with time

**Figure 2:**
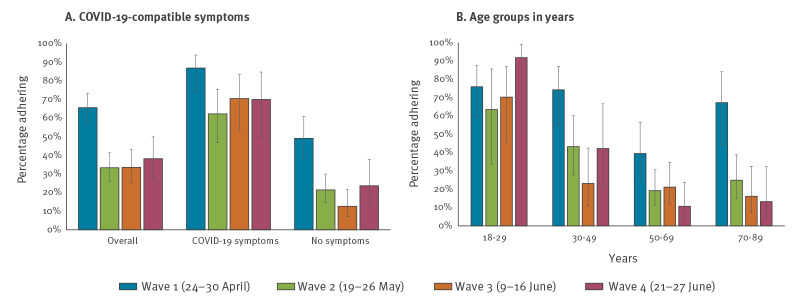
Self-reported adherence to quarantine/isolation over time, (A) overall and by reporting COVID-19-compatible symptoms and (B) by age group, COVID-19 quarantine/isolation study, Norway, 2020

Adherence fell between the first and subsequent waves. In April 2020, the percentage of people who adhered to quarantine/isolation requests was overall 66%, while in waves 2 to 4, adherence varied between 33% and 38% (Figure 2A). The first wave occurred just after the first peak in COVID-19 incidence in Norway when daycare and schools up to grade 4 reopened after having been closed for 5 weeks; the second wave occurred 1 week after schools were fully opened; the third wave occurred when the recommendation on teleworking/working at home was relaxed; the fourth wave occurred during summer holidays when travel to/from several European Economic Area (EEA) countries had just been allowed.

The figure shows weighted percentages and 95% confidence intervals (error bars).

The estimates are calculated for each respective wave with wave-specific weights, as some participants are included in all waves while others participated in fewer waves. The time trend was not tested statistically because of decreasing power and risk of introducing bias. The overall number of individuals who adhered over the total included in the analysis, was n = 86/134 for wave 1, n = 52/178 for wave 3, n = 38/143 for wave 3 and n = 28/106 for wave 4.

The decreasing adherence observed in later waves compared with wave 1 was particularly large among participants who reported no symptoms (Figure 2A). Adherence did not decrease over time among those aged 18–29 years, while it fell among those 50 years of age and older (Figure 2B).

### Ethical statement

The study was approved by the Regional Ethical Committee West (reference number 128391). Participants gave informed consent before completing their first survey.

## Discussion and conclusions

The early results from our ongoing prospective population-based study provide novel information regarding compliance to quarantine/isolation with potential importance for policy formulation.

In Norway, like in many other countries, confirmed and probable COVID-19 cases are required to self-isolate by law [3,5]. Furthermore, household members and close contacts of confirmed cases, and people returning from countries with a COVID-19 incidence of ≥ 20 cases per 100,000 population in the last 2 weeks, are currently (September 2020) obliged to quarantine themselves for 10 days [5,6]. Up to mid-June, all Norwegian residents and visitors entering Norway needed to quarantine. The list of exempted countries is updated weekly [5]. Non-compliance with these regulations can elicit a fine. Fines have been issued and this has been communicated in the press, but there is no proactive follow-up by the police of quarantine/isolation compliance.

We found that adherence to quarantine/isolation in Norway has been low, and especially after the initial surge of infections faded nationwide, and the most drastic physical distancing measures were eased gradually. Our results generate hypotheses that adherence may be influenced by perceived infection risk, or that the population experiences quarantine fatigue and a wish to return to normality. A rapid review of literature on factors affecting adherence to quarantine showed that perceived risk and knowledge of the disease, knowledge about and perceived benefits of quarantine, social norms and practical issues such as financial consequences affected adherence [7]. In our study, we found that adherence was strongly correlated with individuals’ symptoms compatible with COVID-19, as well as their age. Whether better adherence by younger age groups was biased by their lower and decreasing response rate and that therefore mainly those that are health-conscious participated in later waves, or the fact that people in older age groups have fewer contacts [8,9] and might not have defined their lack of contacts as ‘quarantine/isolation’, or whether this is a real effect, is yet unknown. Our findings on age differences were similar to observations made in Italy [10]. Further analyses are required to better understand the determinants of COVID-19 health-related behaviour. We will thus perform a comprehensive analysis of the complete survey data after all six planned waves have been completed.

Irrespective, the overall level of adherence in Norway is so low that, if corroborated, the effect of mandatory quarantine and isolation might be questioned. The fact that our definition of ‘adherence’ is liberal, with only 1 day of completed quarantine/isolation required to be ‘adherent’, makes it likely that we overestimate true adherence. However, all responses were anonymised, so people had little reason to not tell the truth. While invitations to participate in the survey were sampled to represent the Norwegian population concerning age, sex and location, the study population, because of low response rate, had some properties of self-selection, even if supplementary sampling was weighted towards national representativeness. Results may therefore not be fully representative of the Norwegian population.

Despite several limitations, our study results for Norway are cause for concern. Similar data have been collected across a number of European countries, but comparative analyses are not yet published. Initiatives should be considered to better motivate people to engage in behaviours to control the COVID-19 pandemic and respective health communication strategies should be periodically evaluated and adapted. In particular, the Norwegian strategy against the pandemic, expressed as ‘Testing, Isolation, Contact-tracing and Quarantine’ (TISK) needs to be better communicated and reinforced to achieve its aim until a vaccine is available.

## Supplementary Data

512000 Supplement
