# Supplementary material for: Poor self-reported adherence to COVID-19-related quarantine/isolation requests, Norway, April to July 2020
Source: Euro Surveill. 2020 Sep 17;25(37):2001607. doi: 10.2807/1560-7917.ES.2020.25.37.2001607 (PMC7502884; doi:10.2807/1560-7917.ES.2020.25.37.2001607)
Supplement: Supplement [file 20-01607_Supplementarymaterial.pdf]

This supplementary material is hosted by Eurosurveillance as supporting information alongside the article 'Poor self-reported adherence to COVID-19-related quarantine/isolation requests, Norway, April to July 2020', on behalf of the authors, who remain responsible for the accuracy and appropriateness of the content. The same standards for ethics, copyright, attributions and permissions as for the article apply. Supplements are not edited by Eurosurveillance and the journal is not responsible for the maintenance of any links or email addresses provided therein.

SUPPLEMENTARY TABLE. Quarantine/isolation request and adherence, overall and for those who reported to have suffered from COVID-19 compatible symptoms, COVID-19 quarantine/isolation study, Norway, 2020.

		Yes	No	Prefer not to answer or do not know	Totals
**Variable**	**Reported symptoms[a]**	**Number of events with available information**			
Quarantine/isolation requests	Overall	574	3,833	118	4525
	Symptoms	174	918	30	1,122
	No Symptoms	393	2,893	77	3,363
Quarantine/isolation events	Overall	417	4,050	58	4,525
	While reporting a request	204	357	13	574
	Symptoms	198	904	20	1,122
	No Symptoms	217	3,116	30	3,363
Adherence events[b]	Overall	204	357	13	574
	Symptoms	116	54	4	174
	No Symptoms	8	298	8	393
Non-adherence events	Overall	357	204	13	574
	Symptoms	54	116	4	174
	No Symptoms	298	87	8	393
		**Number of participants with available information**			
Quarantine/isolation requests	Overall	402	1,240	62	1,704
	Symptoms	125	523	23	671
	No Symptoms	297	1,093	47	1,437
Quarantine/isolation events	Overall	298	1,375	31	1,704
	Symptoms	148	509	14	671
	No Symptoms	170	1,247	20	1,437
Adherence events[b]	Overall	154	239	9	402
	Symptoms	85	39	1	125
	No Symptoms	75	215	7	297
Non-adherence events	Overall	270	129	3	402

[a] Symptoms that may be compatible with COVID-19 were defined as: fever or high temperature, a cough that has lasted for at least several hours, shortness of breath, aches and pains (e.g. in back, neck, shoulders or joints), blocked nose, sore throat and feeling unusually tired.

[b] Adherence was defined as reporting to have received a request to go into quarantine/isolation for the last 7 days and reported as the percentage having done that at least one day during the period.
